# Castor Oil Seeds

**Published:** 1846-03

**Authors:** 


*Castor Oil Seeds.*—M. Parola detailed at the meeting of Italian savans at Naples, a series of experiments conducted by him to ascertain the best preparation of the castor oil seeds for internal use. The oil itself readily becomes rancid, and neither the syrup nor the emulsion is of general service. M. Parola, founding his statement on numerous chemical and clinical experiments, considers the extract and the ethereal tincture, but especially the latter, as the most certain and most efficacious preparations of the ricinus seeds. He concludes from his experiments: 1st, That the ethereal and alcoholic tinctures have a purgative action four times more powerful than that of the expressed oil, and that they have not a greater tendency to induce vomiting, nor are more irritating than the oil. 2d, That these preparations remain unchanged for a very long time in any climate or weather. 3rdly, That the ethereo-alcoholic extractive principle is comparatively less purgative than the pulp from which it is obtained, showing that the ricinus seeds contain a principle insoluble in alcohol or ether.—*Ibid.*

